# Vallecular Large B-Cell Lymphoma: An Uncommon Presentation of Follicular-Origin Disease

**DOI:** 10.7759/cureus.111607

**Published:** 2026-06-27

**Authors:** Michael J Evans, Amanda M Sonnenburg, Elijah Elliott, Alex L Otto, Kent McIntire

**Affiliations:** 1 College of Osteopathic Medicine, Kansas City University, Joplin, USA; 2 Otolaryngology - Head and Neck Surgery, Freeman Health System, Joplin, USA

**Keywords:** direct microlaryngoscopy, dysphagia, extranodal non-hodgkin lymphoma, follicular large b-cell lymphoma, head and neck lymphoma, vallecula, waldeyer's ring

## Abstract

The vallecula is a mucosal recess of the hypopharynx situated between the base of the tongue and the lingual surface of the epiglottis and bordered laterally by the glossoepiglottic folds. Lesions in this region are uncommon and encompass a broad differential diagnosis, including vallecular cysts, lingual tonsil hypertrophy, minor salivary gland tumors, squamous cell carcinoma, and, rarely, lymphoproliferative disease. Primary extranodal non-Hodgkin lymphoma of the vallecula is exceptionally rare, and follicular large B-cell lymphoma (FLBCL) has not previously been reported at this site. We present the case of a 62-year-old man who presented with progressive dysphagia and persistent globus sensation for 2-3 months. Flexible laryngoscopy demonstrated a well-circumscribed, smooth, cystic-appearing, pedunculated vallecular mass without mucosal ulceration or airway compromise. The patient underwent direct microlaryngoscopy with transoral excisional biopsy. Histopathologic evaluation revealed a high-grade lymphoid neoplasm. Immunophenotyping demonstrated positivity for cluster of differentiation 20 (CD20), cluster of differentiation 10 (CD10), and B-cell lymphoma 6 (BCL6), with co-expression of B-cell lymphoma 2 (BCL2), an elevated Ki-67 proliferation index, and light-chain restriction. Fluorescence in situ hybridization excluded common rearrangements, supporting a diagnosis of CD10-positive LBCL of follicular germinal center origin, classified as FLBCL according to the World Health Organization 5th edition classification of tumors. The patient was treated with rituximab, cyclophosphamide, doxorubicin, vincristine, and prednisone (R-CHOP), followed by involved-site radiotherapy (30 Gy), and achieved a complete metabolic response on surveillance positron emission tomography at six months. The literature on lymphoma involving the vallecula and tongue base is limited to small series and isolated case reports. Diffuse LBCL (DLBCL) predominates, whereas other histologic subtypes have been reported only sporadically. Presentations are typically characterized by dysphagia and globus sensation, and definitive diagnosis requires adequate tissue sampling. To our knowledge, this case represents the first reported occurrence of FLBCL arising in the vallecula. Lymphoma should be considered in the differential diagnosis of atypical or persistent vallecular masses, and adequate submucosal tissue sampling is essential for establishing the diagnosis, particularly in lesions with benign-appearing features.

## Introduction

The valleculae are paired anatomical spaces located between the base of the tongue and the lingual surface of the epiglottis and bounded laterally by the glossoepiglottic folds. Their mucosa is continuous with the lymphoid-rich tissue of the lingual tonsils, with the pre-epiglottic space situated immediately deep to the vallecular floor. Owing to this complex anatomy, masses occupying the vallecular space may arise primarily within the vallecula or extend from adjacent structures, including the tongue base, epiglottis, and supraglottis. As a result, both clinical and radiologic findings are often nonspecific [1].

Most masses occupying the vallecular space are benign, commonly representing mucous retention cysts or prominent lingual lymphoid tissue extending from the tongue base. However, the differential diagnosis also includes minor salivary gland tumors, squamous cell carcinoma of the base of tongue or supraglottis, and lymphoproliferative disorders, particularly when lesions appear submucosal or are associated with cervical lymphadenopathy. Extranodal disease in the upper aerodigestive tract may present with dysphagia, globus sensation, voice changes, or airway symptoms, depending on lesion size and location.

Lymphoma is the third most common head and neck malignancy, following squamous cell carcinoma and salivary gland tumors [2]. Extranodal non-Hodgkin lymphoma involving the oropharynx can be diagnostically challenging because of its variable presentation. Although its overall incidence is increasing, primary involvement of the vallecula remains exceptionally rare [3]. Within Waldeyer’s ring, the base of the tongue accounts for approximately 7% of primary non-Hodgkin lymphomas [4], whereas the vallecula is likely underrepresented because it is frequently grouped with adjacent subsites in the literature.

Follicular large B-cell lymphoma (FLBCL; previously follicular lymphoma grade 3B) is an uncommon germinal center-derived B-cell neoplasm recognized as a distinct entity in the World Health Organization 5th edition classification of tumors. Unlike conventional follicular lymphoma, FLBCL is composed predominantly of large centroblast-like cells. It behaves as an aggressive lymphoma and is managed similarly to diffuse large B-cell lymphoma (DLBCL), the most common B-cell non-Hodgkin lymphoma, rather than as an indolent follicular lymphoma. It is distinguished from DLBCL primarily by its follicular architecture. To our knowledge, FLBCL has not previously been reported arising in the vallecula. We present the case of a 62-year-old man with a vallecular mass ultimately diagnosed as FLBCL and contextualize this finding with a focused review of lymphoma involving the vallecula and base of the tongue, with particular emphasis on the diagnostic and clinical implications for otolaryngologists.

## Case presentation

Histopathology demonstrated squamous mucosa infiltrated by a composite lymphoid proliferation with both follicular and diffuse architectural patterns, composed predominantly of monomorphic large centroblast-like cells with brisk mitotic activity. A targeted immunohistochemical panel demonstrated a strong, diffuse cluster of differentiation 20 (CD20) expression, with cluster of differentiation 10 (CD10) and B-cell lymphoma 6 (BCL6) positivity supporting a germinal center phenotype. B-cell lymphoma 2 (BCL2) was co-expressed, the Ki-67 proliferation index was markedly elevated, and κ/λ light-chain restriction confirmed clonality. Cyclin D1, cluster of differentiation 23 (CD23), and cluster of differentiation 30 (CD30) were negative, excluding mantle cell lymphoma, chronic lymphocytic leukemia/small lymphocytic lymphoma, and classic Hodgkin lymphoma. Because of the high-grade morphology, fluorescence in situ hybridization (FISH) was performed to evaluate for clinically actionable rearrangements. FISH demonstrated BCL2 and dual specificity phosphatase 22-interferon regulatory factor 4 (DUSP22-IRF4) gain/amplification without rearrangements involving v-myc avian myelocytomatosis viral oncogene homolog (MYC), BCL6, or immunoglobulin heavy chain::BCL2 (IGH::BCL2). No DUSP22-IRF4 rearrangement was identified.

Collectively, the morphologic, immunophenotypic, and molecular findings supported a diagnosis of CD10-positive LBCL of follicular germinal center origin, corresponding to FLBCL (previously follicular lymphoma grade 3B) according to the WHO 5th edition classification [5]. The key immunohistochemical and molecular findings are summarized in Table 1.

**Table 1:** Key immunohistochemical and molecular findings supporting the diagnosis CD: cluster of differentiation, BCL2: B-cell lymphoma 2, BCL6: B-cell lymphoma 6, GCB: germinal-center B-cell, CLL/SLL: chronic lymphocytic leukemia/small lymphocytic lymphoma, FISH: fluorescence in situ hybridization, amp: amplification, MYC: v-myc avian myelocytomatosis viral oncogene homolog, IGH: immunoglobulin heavy chain, DUSP22-IRF4: dual specificity phosphatase 22-interferon regulatory factor 4, LBCL-IRF4: large B-cell lymphoma with IRF4 rearrangement.

Marker/study	Result	Interpretive note
CD20	Positive	B-cell lineage
CD10	Positive	Germinal-center origin
BCL6	Positive	Germinal-center origin
BCL2	Positive	Aberrant in the GCB context
Ki-67	Markedly elevated	High proliferative fraction
Cyclin D1/CD23/CD30	Negative	Excludes mantle cell, CLL/SLL, classic Hodgkin lymphoma
κ/λ light chains	Restricted	Confirms clonality
FISH	BCL2 gain/amp; no MYC, BCL6, IGH::BCL2, or DUSP22–IRF4 rearrangement	Excludes high-grade B-cell lymphoma with rearrangements and LBCL-IRF4

Management and outcome 

The patient was referred to medical oncology for staging and systemic therapy. Staging laboratory studies were unremarkable, including a normal complete blood count, lactate dehydrogenase level, comprehensive metabolic panel, and β2-microglobulin level, as well as negative hepatitis B and human immunodeficiency virus screening results.

Staging fluorodeoxyglucose positron emission tomography/computed tomography demonstrated no fluorodeoxyglucose-avid cervical, mediastinal, or distant disease. Bone marrow biopsy showed no evidence of lymphomatous involvement. Based on localized vallecular involvement without nodal, additional extranodal, or bone marrow disease and the absence of B symptoms, the disease was classified as Ann Arbor stage IEA according to the Lugano modification [6]. The International Prognostic Index score was 1 (age >60 years as the sole risk factor), corresponding to low-risk disease [7].

The patient received three cycles of rituximab, cyclophosphamide, doxorubicin, vincristine, and prednisone (R-CHOP), a regimen supported by SWOG 8736 [8] and subsequent positron emission tomography-directed studies, including the Intergroup S1001 trial [9], for limited-stage aggressive BCL. This was followed by involved-site radiotherapy to a total dose of 30 gray (Gy) delivered in 20 fractions of 1.5 Gy each. Interim and end-of-treatment fluorodeoxyglucose positron emission tomography/computed tomography demonstrated a complete metabolic response. At six months after surgical excision and two months after completion of radiotherapy, the patient remained free of disease.

## Discussion

Vallecular lymphomas are exceedingly rare and present a distinct diagnostic challenge for otolaryngologists. Presenting symptoms frequently overlap with those of common benign conditions, such as laryngopharyngeal reflux, vallecular cysts, and lingual tonsil hypertrophy, and the diagnosis is often not initially suspected [1]. Although cross-sectional imaging can be useful for characterizing lesions with suspicious features or uncertain extent, the smooth, cystic appearance of the lesion in the present case, the absence of cervical lymphadenopathy, and the lack of endoscopic evidence of involvement of adjacent structures favored proceeding directly to direct microlaryngoscopy with excision for definitive histopathologic diagnosis. As demonstrated in this case, flexible laryngoscopy may reveal a smooth, cystic-appearing lesion without overt malignant features, highlighting the limitations of endoscopic appearance alone in distinguishing benign from malignant pathology [3]. Given the sparse literature on this subsite, which consists largely of case reports and small case series, we contextualize our findings through a focused review of lymphomas involving the vallecula and base of the tongue.

Epidemiology and subsite distribution 

Oral cavity lymphomas account for approximately 3%-5% of oral malignancies and about 1% of all lymphomas. Most head and neck non-Hodgkin lymphomas arise within lymph nodes, with Waldeyer's ring representing a primary extranodal site in 5%-10% of cases [4]. Within Waldeyer's ring, the palatine tonsils and nasopharynx predominate, whereas the base of the tongue accounts for a smaller proportion, estimated at approximately 7% of primary cases [4].

At the level of the base of the tongue and vallecula, the literature remains limited to small series and isolated case reports. In the largest clinicopathologic series of primary tongue-base lymphoma, only seven cases were identified over a decade, with DLBCL comprising the majority [10]. Vallecular involvement appears to be even less common, with only sporadic reports described to date, including mantle cell lymphoma and the present case of follicular FLBCL [3].

Across reported cases, the subtype distribution broadly parallels that observed in Waldeyer's ring, with B-cell non-Hodgkin lymphoma predominating. DLBCL is the most frequently encountered entity, including presentations with bulky disease and, in some cases, airway compromise [10-12]. Indolent BCL, such as mucosa-associated lymphoid tissue lymphoma and follicular lymphoma, are less commonly reported and may present more subtly, occasionally as incidental findings [13,14]. Mantle cell lymphoma has also been described, typically as a submucosal mass requiring tissue diagnosis [3].

Other subtypes are rare and largely limited to isolated reports. Burkitt lymphoma has only infrequently been described at the base of the tongue [15], whereas peripheral T-cell and extranodal natural killer/T-cell lymphomas, although uncommon, are associated with more aggressive clinical behavior and less favorable outcomes [16,17]. Extranodal Hodgkin lymphoma involving the oropharynx is similarly rare, with only sporadic cases reported, including involvement of the base of the tongue [18]. Overall, while a range of lymphoma subtypes may arise at the base of the tongue and vallecula, DLBCL predominates, with other entities encountered far less frequently.

Clinical presentation patterns 

Symptoms at both subsites are nonspecific and frequently overlap with those of benign mucosal pathology and squamous cell carcinoma. In Waldeyer's ring lymphoma, dysphagia is the most common presenting symptom, followed by neck mass and nasal obstruction, with B symptoms present in a minority of patients [4]. Reported cases involving the base of the tongue and vallecula demonstrate a similar pattern, most often presenting with globus sensation and progressive dysphagia [3,13].

More aggressive presentations, including airway compromise, have been described in cases of bulky disease [12], whereas ulcerative lesions are more frequently associated with Hodgkin and T-cell lymphomas [16,17]. Cystic or partially cystic morphology has also been reported and may be encountered incidentally on imaging, particularly in indolent subtypes [19]. The present case, characterized by a smooth, cystic-appearing, pedunculated lesion associated with globus sensation and dysphagia in the absence of constitutional symptoms, falls within this spectrum, although such morphology may be misleading.

Imaging and endoscopic features 

Imaging findings are generally nonspecific but demonstrate several recurring patterns. Lymphomas involving the base of the tongue and vallecula typically appear on computed tomography as homogeneous, moderately enhancing submucosal masses with relative preservation of the overlying mucosa, in contrast to the ulceration and irregular enhancement more characteristic of squamous cell carcinoma [1]. Larger vallecular lesions may displace the epiglottis posteroinferiorly [3], and cystic morphology has also been described [19].

On magnetic resonance imaging, lesions are typically T2-intermediate and demonstrate diffusion restriction, whereas fluorodeoxyglucose positron emission tomography demonstrates avid uptake and may aid in detecting primary or recurrent disease. Endoscopic findings are variable, ranging from benign-appearing lymphoid hypertrophy to friable masses or ulceration, and are not reliably diagnostic in isolation.

Differential diagnosis and diagnostic approach 

The differential diagnosis for lesions involving the base of the tongue and vallecula is broad. It includes benign processes such as vallecular cysts and lingual tonsil hypertrophy, as well as neoplastic entities, including minor salivary gland tumors and squamous cell carcinoma. Lymphoproliferative disorders remain an important consideration [1]. Clinical features such as a firm submucosal mass, rapid growth, cervical lymphadenopathy, or constitutional symptoms may raise suspicion for lymphoma, although none are pathognomonic.

The present lesion was initially suspected to represent a benign vallecular cyst because of its smooth, cystic appearance, preserved mucosal surface, lack of ulceration, absence of cervical lymphadenopathy, and absence of constitutional symptoms. These features illustrate that lymphoma may closely mimic common benign vallecular pathology and emphasize the importance of maintaining a low threshold for obtaining adequate deep tissue samples from persistent or atypical lesions.

Although cross-sectional imaging may aid in defining lesion extent and identifying features suggestive of malignancy, its use should be individualized based on the clinical presentation and endoscopic findings. In the present case, the lesion appeared smooth and well circumscribed, without mucosal ulceration, cervical lymphadenopathy, or endoscopic evidence of involvement of adjacent structures, favoring direct microlaryngoscopy with transoral excision for definitive histopathologic diagnosis. Potential risks of proceeding without preoperative imaging include underestimation of deep extension, occult involvement of the pre-epiglottic space, and unexpected airway challenges. Accordingly, careful airway assessment and preparedness for alternative airway management strategies are essential when proceeding without preoperative cross-sectional imaging. However, intraoperative inspection confirmed a pedunculated lesion without gross extension, and complete excision was achieved without complication.

Definitive diagnosis depends on adequate tissue acquisition. Fine-needle aspiration is generally insufficient for lymphoma subclassification, and superficial biopsy may be misleading because the lymphoid-rich mucosa may contain normal lingual tonsil tissue that mimics low-grade lymphoid proliferation. Accurate classification requires deeper incisional or excisional sampling with preservation of tissue architecture, ideally extending beneath the lingual tonsil layer. Transoral biopsy with prompt processing for flow cytometry and immunohistochemistry is therefore preferred. Comprehensive immunophenotypic and molecular evaluation is often required, as reliance on routine histology alone may lead to misclassification [3,10].

In cases of significant obstruction, bulky disease, or ulcerative lesions, airway stabilization, including awake fiberoptic intubation or tracheostomy, may be required before biopsy [12,17]. Once a diagnosis is established, staging follows standard lymphoma protocols [6].

Treatment and outcomes 

Management generally follows established lymphoma treatment protocols based on histologic subtype rather than anatomic location. Aggressive BCLs are most commonly treated with R-CHOP, with or without radiotherapy, and have demonstrated favorable responses in reported cases [11]. For limited-stage disease, R-CHOP followed by involved-site radiotherapy is well supported [8,9].

Indolent subtypes may be effectively managed with radiotherapy alone. In contrast, mantle cell lymphoma and T-cell/natural killer-cell lymphomas typically require systemic therapy, with less favorable outcomes reported in the latter group [3,16,17]. Overall, prognosis appears to be driven primarily by histology and stage rather than by anatomic site [10].

Our case in the context of the literature 

This case expands the limited literature on vallecular lymphoma to include a follicular-origin aggressive B-cell entity. Several features align with established patterns, including dysphagia and globus sensation, a smooth submucosal lesion with preserved mucosa, the absence of B symptoms, and a favorable response to R-CHOP followed by involved-site radiotherapy.

Notably, the cystic, pedunculated morphology is atypical, even among the small number of reported cystic lesions at this site [19], and FLBCL has not previously been described in the vallecula. In this case, the lesion was initially suspected to be benign because it was smooth, well circumscribed, mobile, pedunculated, and cystic appearing; lacked mucosal ulceration; and was unaccompanied by cervical lymphadenopathy or constitutional symptoms, features that overlap substantially with those of a simple vallecular cyst. By contrast, features that should raise suspicion for lymphoma at this subsite include a firm or fixed submucosal mass, rapid progression, bulky disease, associated cervical lymphadenopathy, and B symptoms, although none is individually specific. The present case underscores that the absence of such features does not rule out malignancy. A benign-appearing lesion cannot reliably exclude an aggressive lymphoid process, and adequate deep tissue sampling is essential whenever the diagnosis is uncertain.

Management and prognosis 

The otolaryngologist's principal role is to obtain adequate, definitive tissue and, when feasible, as in the present case, to achieve complete excision of a pedunculated lesion that simultaneously relieves obstructive symptoms and secures the diagnosis [2]. Surgery is not curative for lymphoma and is not a substitute for appropriate staging and systemic therapy. Standardized protocols specific to vallecular lymphoma remain lacking because of the rarity of reported cases, reinforcing the importance of early multidisciplinary collaboration and long-term follow-up.

## Conclusions

This case represents the first reported occurrence of FLBCL in the vallecula and highlights several important clinical considerations. A smooth, cystic-appearing vallecular mass should not be presumed benign, and lymphoma should remain within the differential diagnosis. Given the lymphoid-rich nature of the base of the tongue and vallecula, accurate diagnosis typically requires direct microlaryngoscopy with adequate deep or excisional tissue sampling rather than a limited office-based biopsy. Airway planning is also critical and should precede operative intervention in lesions with obstructive potential.

The existing literature on lymphoma involving the base of the tongue and vallecula reflects a diverse but limited spectrum of subtypes, most commonly DLBCL, with other entities reported only sporadically. Presentations are typically characterized by dysphagia and globus sensation, and outcomes are generally favorable in cases of indolent lymphoma and DLBCL when treated with subtype-directed therapy, often including R-CHOP, followed by involved-site radiotherapy for limited-stage disease. Overall, maintaining a low threshold for tissue diagnosis in atypical or persistent vallecular lesions, together with early multidisciplinary coordination, is essential for timely recognition and appropriate management of these uncommon but clinically important presentations. Given the rarity of this entity, the single-case nature of this report, and the relatively short follow-up period, these observations should be regarded as illustrative rather than broadly generalizable.
